# Grey Matter Heterotopia and Criminal Responsibility in a Case of Personal Injury Defense

**DOI:** 10.3389/fpsyt.2020.00261

**Published:** 2020-04-01

**Authors:** Antonietta Curci, Antonio Rampino

**Affiliations:** ^1^Department of Education, Psychology, Communication, University of Bari "Aldo Moro", Bari, Italy; ^2^Department of Basic Medical Science, Neuroscience and Sense Organs University of Bari “Aldo Moro”, Bari, Italy

**Keywords:** Grey Matter Heterotopia, neurodevelopmental disorder, epilepsy, inhibitory control, impulsivity

## Abstract

The abnormal allocation of nodules of grey matter in areas of the brain or spinal cord that should physiologically be occupied by white matter characterizes a neural defect called Grey Matter Heterotopia (GMH). The improvement of MRI techniques has enabled a deeper understanding of the neuropathological bases and epidemiology of such a condition. Among its major manifestations, there is the onset of epileptic seizures, mild intellectual disability, impairments in executive functioning, neurodevelopmental disorders; less frequently GMH has been found associated with depression, anxiety, and schizophrenia. Despite the clinical interest in GMH, no studies have considered the possible forensic and criminological implications of this condition. In the current study, we present a case of GMH in a young male defendant accused of having seriously injured a schoolmate as a reaction to bullying behavior. Neuropsychological and instrumental evidence converge in showing prevalence for the defendant's adoption of repressive responses to stressors, and difficulties to inhibit undesirable behavior at the long run. In the case at hand, the massive stress induced by the exposition to bullying behavior undermined inhibitory control, hence an impulsive and disproportionate reaction took place. Without appropriate therapeutic control, this reactive behavior might take place again. As a consequence, the forensic assessment recommended that the defendant was held partially liable only but that there was likelihood of recidivism. We discuss this single-case evidence for a possible role of GMH in the adoption of dyscontrolled responses to stressors, and the relevance of GMH diagnosis in forensic proceedings.

## Background

Grey Matter Heterotopia (GMH) is a neural defect due to an anomalous development of the cerebral cortex. It is characterized by the presence of small or extended portions of grey matter in areas of the brain or spinal cord that should physiologically be occupied by white matter. Subependymal (periventricular) heterotopia consists of an abnormal allocation of nodules of grey matter within the wall of lateral ventricles in a sub-ependymal position. Studies demonstrate that genetic and epigenetic factors may contribute to the aetiology of this condition. The first include mutations of the X-linked *FNLA* gene (Xq28), coding for the Filamin, an actin-binding protein that crosslinks actin filaments and links actin filaments to membrane glycoproteins. Glycoproteins are involved in remodeling of the cytoskeleton and effect changes in cell shape and migration. Rare mutations or microdeletion of autosomal genes (1, 2) may result further in syndromic disorders. Among epigenetic factors, perinatal stressors, such as hypoxic-ischemic events occurring during migration of neuroblasts, at 7-16 weeks of fetal development, may play a crucial role in the etiopathogenesis of GMH (3).

Neuroimaging techniques have enabled a deeper understanding of the neuropathological bases of GMH, while the epidemiology of such a condition has been widely underestimated in pre-MRI era. For example, rather surprisingly, MRI methodologies have demonstrated that about 15% of brain cortical malformations due to alteration of cortical development are due to GMH and more than 2% of epileptic patients may suffer from GMH (4). Indeed, the major clinical manifestation of GMH is epilepsy. Likely, such a condition emerges from the cytoarchitectural modification that heterotopic migration of neurons determines in grey matter, predictably resulting in a number of electrophysiological modification, including the new formation of epileptogenic foci (5). Along with epilepsy, further manifestations of GMH are represented by mild intellectual disability (ID) and dyslexia, involving impairments in reading ability, processing speed, and executive functioning (4, 6). Moreover, because of the potentially disruptive impact of heterotopic neuronal migration, a number of neurodevelopmental disorders, such as autism (7) and ADHD (8), are associated with GMH. Less frequently, reports of depression (9), anxiety (6), and schizophrenia (1, 10) have been found in comorbidity with GMH (see **Table 1** for a detailed description of the possible neurological and psychiatric comorbidities of GMH).

**Table 1:** List of principal neurological and psychiatric conditions associated with GMH) (1, 4, 5).

PRINCIPAL NEUROLOGICAL CONDITIONS ASSOCIATED WITH GMH
Epilepsy Partial, complex, atypical absence Drop attacks Motor skill loss
**PRINCIPAL PSYCHIATRIC CONDITIONS ASSOCIATED WITH GMH**
Delirium Schizophrenia Schizoaffective Disorder Bipolar Disorder Major Depressive Disorder Autism Spectrum Disorder Attention-Deficits Hyperactivity Disorder Intellectual Disability

To our knowledge, so far none of GMH clinical manifestations has been investigated for its possible associations with forensic and criminological outcomes. However, in children and adolescents with ADHD and other neurodevelopmental disorders, externalizing and antisocial behavioral manifestations might occur, which seldom anticipate more serious criminal behavior in adulthood (11–13). The lack of impulse control that characterizes many of these behaviors poses a challenge for the forensic expert in the evaluation of the criminal liability of an offender and the possible strategies for preventing recidivism in specific cases.

Here we present a case of GMH in a young male defendant (E.L.) accused of having seriously injured a schoolmate after receiving some bullying behavior. We will show here the diagnostic difficulties in the clinical history of E.L. with respect to behavioral and neuropsychiatric manifestations of GMH. We will discuss how these manifestations are related with the committed injury as an issue for the forensic evaluation of the defendant's accountability in criminal trial and prevention of possible recidivism.

## Case Presentation

E.L. is an 18-year-old Caucasian male, who is accused of having severely wounded another student in a scuffle after school two years earlier. Due to the physical conflict, E.L.'s schoolmate reported an injury of his left eyeball, and E.L. was charged with causing serious personal injuries.

E.L. has a complex clinical history. Born at term of natural childbirth, he was artificially fed, and his development followed regular phases until the age of 2. In the course of subsequent months, first symptoms of a delay in language development emerged, and, at the age of 4, E.L. was diagnosed with an Autism Spectrum Disorder (ASD). While attending the first years of primary school, he showed good interactions with peers, though experiencing significant learning difficulties. According to his parents' accounts and consistent with school reports, sometimes E.L. appeared restless and hyperactive. Clinical assessment identified mild cognitive disability associated with language disorder, subsequently revised as learning disability associated with affective immaturity and scarce tolerance to frustrations. At the age of 8, E.L. was hospitalized for re-assessment of his diagnosis, which was indeed revised as a developmental mixed disorder with a major impairment of linguistic capacity. In the same period, E.L. experienced epileptiform seizures. At the age of 9, following a MRI, he was diagnosed with Grey Matter Heterotopia, while first problems of socialization appeared alongside: E.L. became a target of his peer social pressure and bullying to the extent that he decided to move school the year after.

E.L. has a mild and submissive temperament, particularly wishful to establish meaningful friendship relationships, a trait of his personality that has paradoxically backfired with him becoming the object of bad jokes and mistreatments by his classmates and acquaintances. He has a very supportive family, with parents thoughtfully caring for his health and social concerns. Over the years, E.L. has undergone further diagnostic refinements, which progressively evidenced depressive tendencies and social withdrawal along with linguistic and cognitive disabilities and developmental emotional disorder. In medical records, the condition of E.L. is described as characterized by attention and memory lability especially in the school context, motor instability, affective immaturity, and difficulty in emotion regulation and inhibition (F80.8, F81.9, F93 ICD10). Within the overall picture, however, the clinical implications of GMH are left almost completely unconsidered except for their role in the emergence of epileptic seizures.

The troubles in adaptation and socialization led E.L. to experience severe episodes of social rejection and bullying, which made him feel profoundly sad and worthless. As an aggravation of this psychological condition, at the age of 16, seven months before the dispute with his schoolmate, he engaged in a suicidal attempt by ingesting an overdose of sleeping pills. He was hospitalized with the diagnosis of depression in a passive-dependent personality and thought disorders, started on antipsychotic treatment (aripiprazole, 10 ml per day). The therapeutic program increased his behavioral control, but negatively affected personal interests, energy, and socialization, so that the antipsychotic dosage was progressively reduced until complete discontinuation, which was reached a couple of months before the criminal conduct he was later charged with. On the day of the alleged offence, E.L. had a verbal dispute with one of his classmates for a trivial reason, and received verbal abuse in return. On leaving the school, E.L. found that girl along with her boyfriend and other schoolmates, waiting for him beyond the school gate threateningly staring at him. Frightened by the scene, E.L. tried to escape running as fast as he could, but he was intercepted and stopped by the boyfriend of his classmate who roughly blamed him for the dispute he had had with the girl few hours before. A scuffle ensued, and the two boys fell to the ground, with E.L. punching blindly a break through and free himself from people around him. Following the fight, E.L. reported small facial and knee injuries while his antagonist was diagnosed with an outbreak of his left eyeball. Shocked by the unpredictable evolution of the events, E.L. became progressively more aware of the severity of the injuries he had inflicted. Very soon, he started to feel guilty and became regretful. He repeatedly declared he had never intended to harm his mate so severely and explained his exclusive aim had been to escape the frightened situation he had found himself in. Rather interestingly, he also displayed an almost complete amnesia for the event and his running off.

E.L. is submitted to expert examination following a Court order to establish his criminal liability at the time of the facts and the actual risk of recidivism. The psycho-forensic assessment included clinical interviews and test administration, the results of which are displayed in **Table 1**. Furthermore, E.L. underwent a structural Magnetic Resonance Imaging (sMRI) scan to ascertain the presence of a previously reported area of heterotopic grey matter in E.L.'s brain. MRI was performed on a GE Signa 3T scanner (GE Healthcare).

As **Table 2** shows, E.L. appears to have a normal level of intelligence with mild impairment in processing speed and receptive linguistic capacities. He appears suspicious, with persistent persecutory thought contents, phobic ideas, and obsessive worries regarding the future. E.L. preferably adopts a defensive modality of coping with anger expression, with a dominance of repressive and avoidant tendencies. His affective state is dysphoric and characterized by high levels of anxiety. The neuropsychological assessment reveals a poor executive capacity of inhibiting dominant responses, set shifting, planning, and strategic control. However, E.L. appears able to choose and manage effective problem solving strategies, which do not imply space or time constraints. Finally, a low risk propensity is observed. The psychometric conclusions are consistent with evidence obtained through the clinical interviews.

**Table 2:** Psychometric evaluation of E.L.

Test	Score
TROG-2 (standard scores)	95
WAIS-IV (standard scores)	IQ = 90; VC = 74; PR = 112; WM = 103; PS = 81
MMPI-2 (T-scores)	Pa = 68; Pt = 68; Hs = 63; D = 60
STAI-Y (T-scores)	Y-1 = 90 Y-2 = 70
STAXI (T-scores)	S-Anger = 49; T-Anger < 34; AX/In = 44; Ax/Out = 44; Ax/Con = 57; Ax/EX = 37
BDI-II	15
FF (cut-off = 6)	2.7
TMT A-B (cut-off: A = 45”, B = 122”)	A = 62”, B = 145”
P-M (cut-off = 15.5”)	86”
ToL (cut-off = 29)	14
EPMT (standard scores)	113.8
WCST (standard scores)	100-108
BART (cut-off: Orange = 3.5; Yellow = 12.3; Blue = 33.3)	Orange = 2.77; Yellow = 3.67; Blue = 7.7

Standard scores, T-scores, and cut-off were set-up according to Italian norms, when available. TROG-2, Test for Reception of Grammar-2 (14); WAIS-IV, Wechsler Adult Intelligence Scale IV; VC, Verbal Comprehension; PR, Perceptual Reasoning; WM, Working Memory; PS, Processing Speed (15); MMPI-2, Minnesota Multiphasic Personality Inventory 2 (16); STAI-Y, State-Trait Anxiety Inventory (17); STAXI, State-Trait Anger Inventory (18); BDI-II, Beck Depression Inventory-II (19); FF, Phonemic Fluency (20); TMT A-B, Trail Making Test A and B (21); P-M, Plus-Minus Test (22); ToL, Tower of London (23); EPMT, Elithorn Perceptual Maze Test (24); WCST, Wisconsin Card Sorting Test (25); BART, Balloon Analogue Risk Task (26).

**Figure 1:**
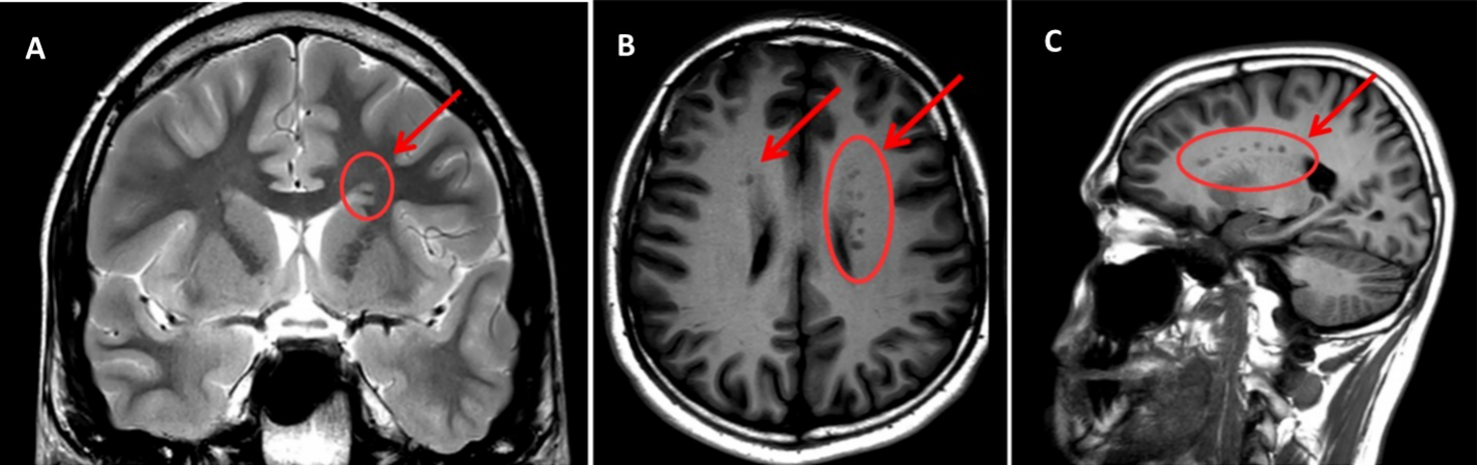
MRI scan sections of the patient's brain reporting the presence of GMH (indicated by arrows and circles). **(A)** T2W – coronal section; **(B)** T1W- assial section; **(C)** T1W – sagittal section. Arrows indicate areas of subependimal alterated signal (bilateral and asymmetrical PNH—Periventricular Nodular Heterotopia—with heterotopic grey matter stretching all along the ventricular walls, maximum diameter = 5 mm). Note that the shade of grey is the same as that of the cortical grey matter (the same signal intensity), which confirms that it is grey matter—the pathognomonic finding in GMH.

The outcome of sMRI scanning confirms previous evidence of a sub-ependymal GMH in E.L.'s brain, excluding the presence of other morphological and/or structural abnormalities (**Figure 1**).

## Discussion

Following the clinical and instrumental examination, the expert opinion in the criminal proceeding recommended partial liability of the defendant: E.L. appears able to understand the real implications and moral wrongfulness of his action against his schoolmate. In fact, he asks for forgiveness to the victim. His cognitive capacity is intact, as long as his problem solving ability when in absence of space or time constraints. However, relevant impairments result in his executive capacities, in that he appears unable to inhibit emotionally overwhelming and compelling responses, with difficulties in set shifting, planning, and strategic control. As a consequence, under particular circumstances, E.L.'s behavior is driven by impulsivity to the extent that, once started, it cannot readily be stopped. From the clinical point of view, anxiety and phobias are the affective responses to external overwhelming pressure. Overall, the clinical and neuropsychological pattern emerging from the examination of E.L. appears compatible with the clinical manifestations of GMH (4).

During the course of his development, E.L. has learnt to adjust to his executive impairment, by adopting a behavioral risk control. In other words, being somewhat aware of his executive deficits, he prevents fatal consequences through low engagement in defiance and challenges. The dispute in the classroom and the ensuing consequences with the schoolmates are some of those particular circumstances in which E.L. could have experienced a failure in impulse control. In an attempt to desperately run from the threatening situation, he engaged in a sequence of actions that exceeded the limits of a reasonable reaction. Although able to understand the inappropriateness of his conduct, E.L. is unable to exert a full control on it. At the time of the alleged offence, E.L. had suspended the therapeutic treatment. The action flowed unwillingly, without restraints, so that E.L. cannot be considered completely responsible for that (27).

Anamnestic data suggest that E.L. has a mild and submissive temperament, and psychometric assessment indicates a prevalent adoption of repressive modalities of response to stressors. In other words, E.L. appears usually able to inhibit and control undesirable behavior. However, inhibition is a resource-consuming process, highly vulnerable when self-regulatory capacity is reduced by prior exertion of self-control. Ego-depletion theory postulates that the self has a limited amount of resources, used for a wide range of tasks, such as emotion regulation, reasoning, decision making, impulse control, behavioral performance, and so on (28). In case of a low availability of executive resources, a massive stress can induce a significant impairment on inhibitory control, hence concomitant actions can develop in all their disruptiveness. Although auto-directed, the suicidal behavior that E.L. manifested a few months before the scuffle is indicative of such a failure.

### Possible Role of GMH in E.L. Behavior

Brain morphological (MRI) assessments on E.L. were performed in three different stages of his life: once during his early childhood, once during his teenage years, and last time during the forensic assessment. All assessments concluded for the presence of a GMH area within the wall of one lateral ventricule in E.L.'s brain.

With regard for the events that brought E.L. to be accused of severe injuries against his schoolmate, the MRI findings may be of critical relevance. In fact, while studies have reported that GMH is associated with seizure emergence, that is also observed in the case for E.L., there is consistent evidence that epilepsy, in turn, is associated with impulsive and potentially harmful behaviors. In fact, studies report that aggression and impulsivity are observed in a minority of people with epilepsy, sometimes occurring as a part of a more complex psychiatric comorbidity, sometimes also emerging as a side effect of anticonvulsant agents. In line with these reports, other studies have shown that epilepsy and impulsive behavior share common neurotransmitter systems and brain regions, including the GABA, glutamate, serotonin, dopamine, and noradrenaline systems and the hippocampus, amygdala, prefrontal cortex, anterior cingulate cortex, and temporal lobes (29). On the other hand, there are studies supporting the evidence that GMH *per se* is associated with impulsivity and aggressive behaviors. In particular, heterotopic grey matter presence is found associated with aggression, impulsive violence, inability to control anger and lack of remorse violent acts (1, 6, 30, 31).

In the case we here report, it is possible that psychological features above described interact with brain structure and electrophysiological alteration in reducing impulse control and increasing intolerance to emotionally overwhelming stimulation, resulting in the overall inability to establish appropriate and effective behavioral responses. These responses are instead substituted with chaotic and aggressive behavioral patterns. The present considerations, although non-definitive on a causal role of GMH on the pathogenesis of criminal behavior, might be useful when evaluating the defendant's impulsivity in forensic contexts. More generally, the present case exemplifies the need to implement neuroscientific evidence in forensic assessment of violent and dyscontrolled behavior of forensic relevance. The presence of a neural substrate for a psychopathological condition contributes to explain the difficulties of some forensic patients in inhibiting impulsive criminal acts, so that their responsibility is diminished. Neuroscientific assessment – following the Daubert's standards (Daubert v. Merrell Dow Pharmaceuticals, 509 U.S. 579, 1993) –can thus contribute to the legal decision of judges and juries on the criminal responsibility of defendants under the BARD (Beyond-Any-Reasonable-Doubt) criteria (32). Additionally, the convergence between neuropsychological and instrumental evidence has implications on prevention of recidivism in cases similar to that here described, by suggesting that, under acute pressure and without adequate therapeutic control, impulsive and disproportionate reactions to hostile actions might take place again.

## Data Availability Statement

The data sets generated for this study are available on request to the corresponding author.

## Ethics Statement

Ethical approval was not provided for this study because data were collected following a request of a Criminal Court of providing an expert opinion on a case of personal injury defense. Written informed consent was obtained from the participant for the publication of any potentially identifiable images or data included in this article.

## Author Contributions

AC wrote the second, third, and fourth paragraphs of *Background*; *Case Presentation* except the last two paragraphs; *Discussion* except *Possible Role of GMH in E.L. Behavior*, and set up **Table 2**. AR wrote the first paragraph of *Background*, the last two paragraphs of *Case Presentation*, *Possible Role of GMH in E.L. Behavior*, and set up **Table 1** and **Figure 1**. Both authors have made substantial, direct, and intellectual contributions to the work, and approved it for publication.

## Funding

Publication of the study was supported by grant no. 86602-VIII/2 of the University of Bari "Aldo Moro".

## Conflict of Interest

The authors declare that the research was conducted in the absence of any commercial or financial relationships that could be construed as a potential conflict of interest.
